# Harmonisation of in-silico next-generation sequencing based methods for diagnostics and surveillance

**DOI:** 10.1038/s41598-022-16760-9

**Published:** 2022-08-23

**Authors:** J. Nunez-Garcia, M. AbuOun, N. Storey, M. S. Brouwer, J. F. Delgado-Blas, S. S. Mo, N. Ellaby, K. T. Veldman, M. Haenni, P. Châtre, J. Y. Madec, J. A. Hammerl, C. Serna, M. Getino, R. La Ragione, T. Naas, A. A. Telke, P. Glaser, M. Sunde, B. Gonzalez-Zorn, M. J. Ellington, M. F. Anjum

**Affiliations:** 1 Animal and Plant Health Agency (APHA), Weybridge, UK; 2 Wageningen Bioveterinary Research (WBVR), Lelystad, The Netherlands; 3 Universidad Complutense de Madrid (UCM), Madrid, Spain; 4 Norwegian Veterinary Institute (NVI), Oslo, Norway; 5 Public Health England (PHE), London, UK; 6 Agence nationale de sécurité sanitaire de l’alimentation, de l’environnement et du travail (ANSES), Unité Antibiorésistance et Virulence Bactériennes, Maisons-Alfort, France; 7 German Federal Institute for Risk Assessment (BfR), Berlin, Germany; 8 University of Surrey (UoS), Guildford, UK; 9 Institute Pasteur, EERA Unit, Paris, France; 10 Assistance Publique Hopitaux de Paris, Paris, France

**Keywords:** Data processing, Molecular medicine, Antimicrobial resistance

## Abstract

Improvements in cost and speed of next generation sequencing (NGS) have provided a new pathway for delivering disease diagnosis, molecular typing, and detection of antimicrobial resistance (AMR). Numerous published methods and protocols exist, but a lack of harmonisation has hampered meaningful comparisons between results produced by different methods/protocols vital for global genomic diagnostics and surveillance. As an exemplar, this study evaluated the sensitivity and specificity of five well-established in-silico AMR detection software where the genotype results produced from running a panel of 436 *Escherichia coli* were compared to their AMR phenotypes, with the latter used as gold-standard. The pipelines exploited previously known genotype–phenotype associations. No significant differences in software performance were observed. As a consequence, efforts to harmonise AMR predictions from sequence data should focus on: (1) establishing universal minimum to assess performance thresholds (e.g. a control isolate panel, minimum sensitivity/specificity thresholds); (2) standardising AMR gene identifiers in reference databases and gene nomenclature; (3) producing consistent genotype/phenotype correlations. The study also revealed limitations of in-silico technology on detecting resistance to certain antimicrobials due to lack of specific fine-tuning options in bioinformatics tool or a lack of representation of resistance mechanisms in reference databases. Lastly, we noted user friendliness of tools was also an important consideration. Therefore, our recommendations are timely for widespread standardisation of bioinformatics for genomic diagnostics and surveillance globally.

## Introduction

Next Generation Sequencing (NGS), a DNA sequencing technology, has become an established technique with hundreds of publications each year detailing the use and advancement of this technology, often replacing other gene-based typing tools such as PCRs, and microarrays^1–3^. Furthermore, NGS high-throughput platforms, which in recent years have seen radical improvements in quality, running times and cost, have revolutionised the diagnosis of health-related issues in animals and humans. This includes infectious disease diagnosis, where in-silico (or computer based) genetic data analysis is aiding and, in some cases, substituting more complex and costly laboratory techniques^4^. The COVID-19 pandemic is a testimony of the usefulness of this technology for both research and surveillance^5,6^. Similarly, by using NGS technologies such as sequencing the whole genome of bacterial isolates, transmission chains of pathogens and a global overview of their population structure is being identified, helping inform surveillance and to trace outbreaks^7^. For antimicrobial resistance (AMR) detection in bacteria, which is another global threat leading to decreasing therapeutic options and increasing treatment failures, antimicrobial susceptibility testing which produces a phenotype, is progressively being substituted by detection of the underlying genetic mechanisms using whole genome sequencing (WGS) of bacterial isolates. Nevertheless, performing correlations between pheno- and geno-types remains essential, as phenotypes are still accepted as the gold standard due to genotypes being based only on already known AMR genes so new variants may be missed. WGS analysis also facilitates the identification of bacteria such as *Escherichia coli*, their lineage, and plasmids, in addition to genetic features such as resistance to critically important antimicrobials for therapeutics, and chromosomal mutations, deletions and insertions that may be associated with AMR phenotypes^8–13^. Characterisation of bacterial plasmids, which often transfer AMR genes due to their mobility, is particularly important and has become more achievable by combining short and long read WGS so complete AMR plasmid genomes can be determined^14–16^.

However, a major barrier to the application of bioinformatics software for AMR detection beyond individual research applications, to diagnosis and national and/or international surveillance, is the standardisation of both DNA-based laboratory techniques and in-silico analysis. The availability of a plethora of bioinformatics tools and pipelines, with continual rapid advancement in this area, has resulted in no standardised methodology or nomenclature making comparisons across compartments (e.g. humans, animals, and environment) or institutes difficult. In 2019, Hendriksen et al.^17^ reported at least 47 bioinformatics tools were freely available, and no doubt this has increased further. However, to understand the epidemiology of AMR in an One-Health context, it is vital to harmonise in-silico AMR detection methods, as has been established for bacteria such as Methicillin-resistant *Staphylococcus aureus* (MRSA), where ideal requirements for molecular typing techniques have been clearly defined^18,19^. There have been similar discussions for in-silico AMR detection^20^, and although the recommendations have not been properly evaluated, a small multi-centre study with nine institutes that performed predictions of AMR genotypes from 10 samples harbouring carbapenem-resistant organisms, showed that differences in the database selected and gene coverage thresholds were some of the factors contributing to variation in AMR results^21^. Such evaluations are required at a much larger scale because supranational organisations such as the World Health Organisation (WHO), European Centre for Disease Control (ECDC) and the European Food Safety Authority (EFSA) have recommended the use of genomics within international surveillance programmes that compare AMR trends across countries in Europe and worldwide, to help tackle the spread of multi- and extensive drug resistant bacteria which are the cause of great concern^22–24^.

In-silico AMR detection methods are based on a three-step process: i) sample preparation such as bacterial culture and DNA extraction; ii) whole genome sequencing; and iii) in-silico analysis of data produced from isolate WGS. This process offers attractive possibilities for diagnostic test automation, including parallelising tests for multiple characteristics and even retrospective exploration for novel AMR genotypes without having to repeat steps i) or ii). AMR detection pipelines are based on existing knowledge of AMR genotype–phenotype associations^25^.

To detect the genotype or underlying genetic mechanism for resistance by screening the WGS data obtained from isolated bacteria, the bacterial DNA is compared against a reference set of DNA sequences, also known as a database (normally in FASTA or text-based format) containing the genotypes (i.e. AMR genes or point mutations) responsible for known AMR phenotypes.

Bacterial DNA can be compared against the database using two different techniques: either by mapping the WGS short-reads onto the reference DNA sequences in the database, or by basic local alignment search^26^ using the assembled genome contigs as a query against the database. While the first approach may be faster (< 10 min for a single core computer) and straight forward, it involves dealing with large raw data files (e.g., paired short-reads raw data files of up to 300 Mbytes for a 5 Mbase genome such as *Escherichia coli*). The second method may require a longer running time (~ 25 min) as the raw data files must be de novo assembled prior to comparison against the database. Many laboratories perform the assembly step as a routine for other purposes, so the extra running time might not be a burden. Once assembly for the bacterial genome has been stored, re-running the pipeline to screen for a novel AMR gene should be a matter of a few seconds.

As part of the One-Health European Joint Programme Project ARDIG (Antibiotic Resistance Dynamics: the influence of geographic origin and management systems on resistance gene flows within humans, animals and the environment)^27^, nine partners compared the software performance of five pipelines for AMR detection based on WGS of *E. coli* isolates, and this paper describes that work. Each software detected the presence or absence of genes and point mutations associated with the sensitivity phenotype to 14 antimicrobials established for all isolates, which were taken as the “gold-standard”. This study restricted its scope to comparing the performance of pipelines in terms of their sensitivity and specificity to detect AMR, under the default settings defined by the pipelines’ authors. No attempt to evaluate the software installation process was considered in this study since most of the users agreed that IT support is provided institutionally. Isolate DNA extraction and sequencing protocols used by participating institutes were also not evaluated in this study.

## Results

### Antimicrobial sensitivity of *E. coli*

A total of 436 *E. coli* collected by nine different collaborating institutes working in the veterinary and human health sectors in Europe, were included in the study. Table 1 provides overview of the isolates, including year of isolation and percentage from each reservoir and country, with full details provided in Methods and Supplementary Table S1. The antimicrobial susceptibilities of all isolates were established to a panel of 14 antimicrobials; these were used as the gold standard for the study and are given for each isolate in Supplementary Table S1, with the number of resistant, in comparison to sensitive isolates, provided in Table 2.

**Table 1:** Distribution of the 436 *E. coli* isolates by year, source, and country.

Years	Counts	Percentage	Source	Counts	Percentage	Country	Counts	Percentage
2006	1	0.23	Beef	2	0.46	France	98	22.48
2007	5	1.15	Broiler	86	19.72	France (Polynesia)	1	0.23
2008	4	0.92	Cattle	51	11.7	Germany	50	11.47
2009	3	0.69	Chicken meat	14	3.21	Netherlands	50	11.47
2010	2	0.46	Dog	9	2.06	Norway	50	11.47
2011	4	0.92	Goose	1	0.23	Spain	50	11.47
2012	9	2.06	Gull	5	1.15	UK	137	31.42
2013	10	2.29	Horse	1	0.23			
2014	26	5.96	Human	150	34.4			
2015	118	27.06	Pig	88	20.18			
2016	48	11.01	Pork	7	1.61			
2017	71	16.28	Rabbit	1	0.23			
2018	77	17.66	Red fox	10	2.29			
2019	58	13.3	Turkey	1	0.23			
			Turkey meat	1	0.23			
			Wild bird	9	2.06			

All human isolates were of clinical origin, whilst the animal isolates were from healthy animals; meat isolates were assumed to be from healthy animals as they had entered the food chain.

**Table 2:** Number of resistant and susceptible isolates per antimicrobial used in this study.

Institute	# Strains	Ampicillin (res/sen)	Azithromycin (res/sen/missing MIC)	Cefotaxime (res/sen)
APHA	37	33 (89.19%)/4 (10.81%)	7 (18.92%)/30 (81.08%)	17 (45.95%)/20 (54.05%)
BfR	50	41 (82.0%)/9 (18.0%)	5 (10.0%)/45 (90.0%)	28 (56.0%)/22 (44.0%)
ANSES	49	49 (100.0%)/0 (0.0%)	10 (20.41%)/39 (79.59%)	49 (100.0%)/0 (0.0%)
UCM	50	39 (78.0%)/11 (22.0%)	3 (6.0%)/47 (94.0%)	0 (0.0%)/50 (100.0%)
UoS	50	49 (98.0%)/1 (2.0%)	32 (64.0%)/18 (36.0%)	47 (94.0%)/3 (6.0%)
Pasteur	50	42 (84.0%)/8 (16.0%)	15 (30.0%)/35 (70.0%)	28 (56.0%)/22 (44.0%)
WBVR	50	32 (64.0%)/18 (36.0%)	0 (0.0%)/50 (100.0%)	24 (48.0%)/26 (52.0%)
PHE	50	50 (100.0%)/0 (0.0%)	0 (0%)/0 (0%)/50*	43 (86.0%)/7 (14.0%)
NVI	50	44 (88.0%)/6 (12.0%)	0 (0%)/0 (0%)/50*	29 (58.0%)/21 (42.0%)
Total	436	379 (86.93%)/57 (13.07%)	72 (16.51%)/264 (60.55%)/100*	265 (60.78%)/171 (39.22%)

Institute	Ceftazidime (res/sen)	Chloramphenicol (res/sen/missing MIC)	Ciprofloxacin (res/sen)	Colistin (res/sen)
APHA	16 (43.24%)/21 (56.76%)	18 (48.65%)/19 (51.35%)	22 (59.46%)/15 (40.54%)	0 (0.0%)/37 (100.0%)
BfR	29 (58.0%)/21 (42.0%)	19 (38.0%)/31 (62.0%)	29 (58.0%)/21 (42.0%)	18 (36.0%)/32 (64.0%)
ANSES	43 (87.76%)/6 (12.24%)	27 (55.1%)/22 (44.9%)	36 (73.47%)/13 (26.53%)	7 (14.29%)/42 (85.71%)
UCM	0 (0.0%)/50 (100.0%)	16 (32.0%)/34 (68.0%)	15 (30.0%)/35 (70.0%)	2 (4.0%)/48 (96.0%)
UoS	45 (90.0%)/5 (10.0%)	3 (6.0%)/47 (94.0%)	44 (88.0%)/6 (12.0%)	0 (0.0%)/50 (100.0%)
Pasteur	13 (26.0%)/37 (74.0%)	7 (14.0%)/43 (86.0%)	19 (38.0%)/31 (62.0%)	0 (0.0%)/50 (100.0%)
WBVR	24 (48.0%)/26 (52.0%)	12 (24.0%)/38 (76.0%)	16 (32.0%)/34 (68.0%)	0 (0.0%)/50 (100.0%)
PHE	41 (82.0%)/9 (18.0%)	0 (0%)/0 (0%)/50*	50 (100.0%)/0 (0.0%)	0 (0.0%)/50 (100.0%)
NVI	29 (58.0%)/21 (42.0%)	11 (22.0%)/39 (78.0%)	30 (60.0%)/20 (40.0%)	0 (0.0%)/50 (100.0%)
Total	240 (55.05%)/196 (44.95%)	113 (25.92%)/273 (62.61%)/50	261 (59.86%)/175 (40.14%)	**27 (6.19%)/409 (93.81%)**

Institute	Gentamicin (res/sen)	Meropenem (res/sen/missing MIC)	Nalidixic Acid (res/sen/missing MIC)	Sulfamethoxazole (res/sen/missing MIC)
APHA	8 (21.62%)/29 (78.38%)	0 (0.0%)/37 (100.0%)	20 (54.05%)/17 (45.95%)	32 (86.49%)/5 (13.51%)
BfR	11 (22.0%)/39 (78.0%)	0 (0.0%)/50 (100.0%)	25 (50.0%)/25 (50.0%)	35 (70.0%)/15 (30.0%)
ANSES	21 (42.86%)/28 (57.14%)	0 (0.0%)/49 (100.0%)	38 (77.55%)/11 (22.45%)	44 (89.8%)/5 (10.2%)
UCM	6 (12.0%)/44 (88.0%)	0 (0.0%)/50 (100.0%)	28 (56.0%)/22 (44.0%)	36 (72.0%)/14 (28.0%)
UoS	12 (24.0%)/38 (76.0%)	0 (0.0%)/50 (100.0%)	40 (80.0%)/10 (20.0%)	45 (90.0%)/5 (10.0%)
Pasteur	12 (24.0%)/38 (76.00%)	23 (46.0%)/27 (54.0%)	20 (40.0%)/30 (60.0%)	39 (78.0%)/11 (22.0%)
WBVR	0 (0.0%)/50 (100.0%)	0 (0.0%)/50 (100.0%)	8 (16.0%)/42 (84.0%)	26 (52.0%)/24 (48.0%)
PHE	21 (42.0%)/29 (58.0%)	26 (52.0%)/24 (48.0%)	0 (0%)/0 (0%)/50*	0 (0%)/0 (0%)/50*
NVI	7 (14.0%)/43 (86.0%)	0 (0.0%)/45 (100.0%)/5*	18 (36.0%)/32 (64.0%)	29 (58.0%)/21 (42.0%)
Total	98 (22.48%)/338 (77.52%)	49 (11.24%)/382 (87.61%)/5*	197 (45.18%)/189 (43.35%)/50*	286 (65.6%)/100 (22.94%)/50*

Institute	Tetracycline (res/sen/missing MIC)	Tigecyline (res/sen/missing MIC)	Trimethoprim (res/sen/missing MIC)	
APHA	32 (86.49%)/5 (13.51%)	0 (0.0%)/37 (100.0%)	29 (78.38%)/8 (21.62%)	
BfR	38 (76.0%)/12 (24.0%)	0 (0.0%)/50 (100.0%)	33 (66.0%)/17 (34.0%)	
ANSES	42 (85.71%)/7 (14.29%)	0 (0.0%)/49 (100.0%)	36 (73.47%)/13 (26.53%)	
UCM	45 (90.0%)/5 (10.0%)	0 (0.0%)/50 (100.0%)	34 (68.0%)/16 (32.0%)	
UoS	28 (56.0%)/22 (44.0%)	0 (0.0%)/50 (100.0%)	38 (76.0%)/12 (24.0%)	
Pasteur	22 (44.0%)/28 (56.0%)	0 (0.0%)/50 (100.0%)	23 (46.0%)/27 (54.0%)	
WBVR	25 (50.0%)/25 (50.0%)	0 (0.0%)/50 (100.0%)	14 (28.0%)/36 (72.0%)	
PHE	0 (0%)/0 (0%)/50*	1 (2.0%)/49 (98.0%)	0 (0%)/0 (0%)/50*	
NVI	31 (62.0%)/19 (38.0%)	0 (0.0%)/45 (100.0%)/5*	20 (40.0%)/30 (60.0%)	
Total	263 (60.32%)/123 (28.21%)/50*	1 (0.23%)/430 (98.62%)/5*	227 (52.06%)/159 (36.47%)/50*	

The numbers, including percentage, of resistant and susceptible isolates provided by the nine collaborating institutes for each of the 14 antimicrobials are given. Antimicrobials for which the MIC data was not available for all, or part of the isolates provided by an Institute have been marked (*). Cells in bold indicate unbalanced ratio with resistant isolates being less than 10% of total.

Our panel of *E. coli* did not show an even distribution of resistance for all 14 antimicrobials, although for most antimicrobials the numbers of resistant isolates in comparison to sensitive ones was more than 10% of the total, so enabled accurate sensitivity estimates to be made (Table 2). However, smaller numbers of isolates were determined as resistant to colistin (n = 27 or 6.2%) and tigecycline (n = 1 or 0.23%), consistent with the scarcity of *E. coli* resistant to these high priority critically important antimicrobials (HP-CIAs) in Europe. The unbalanced ratio between resistant and susceptible isolates for these antimicrobials will result in loss of precision for the sensitivity estimates. Nevertheless, sensitivity estimates for colistin was still calculated but for tigecycline, the sensitivity was not calculated due to the very low numbers of resistant isolates.

### Pipeline comparisons

Five pipelines were selected for testing: GeneFinder^28^; APHA SeqFinder/Abricate^14^; WBVR BLAST (in-house pipeline); ResFinder/PointFinder^29,30^; ARIBA^31^. Some differences existed between the pipelines in their data input method or the algorithm used for detecting gene presence, which were part of the criteria for selecting these pipelines (see Methods and Supplementary Table S2).

**Figure 1:**
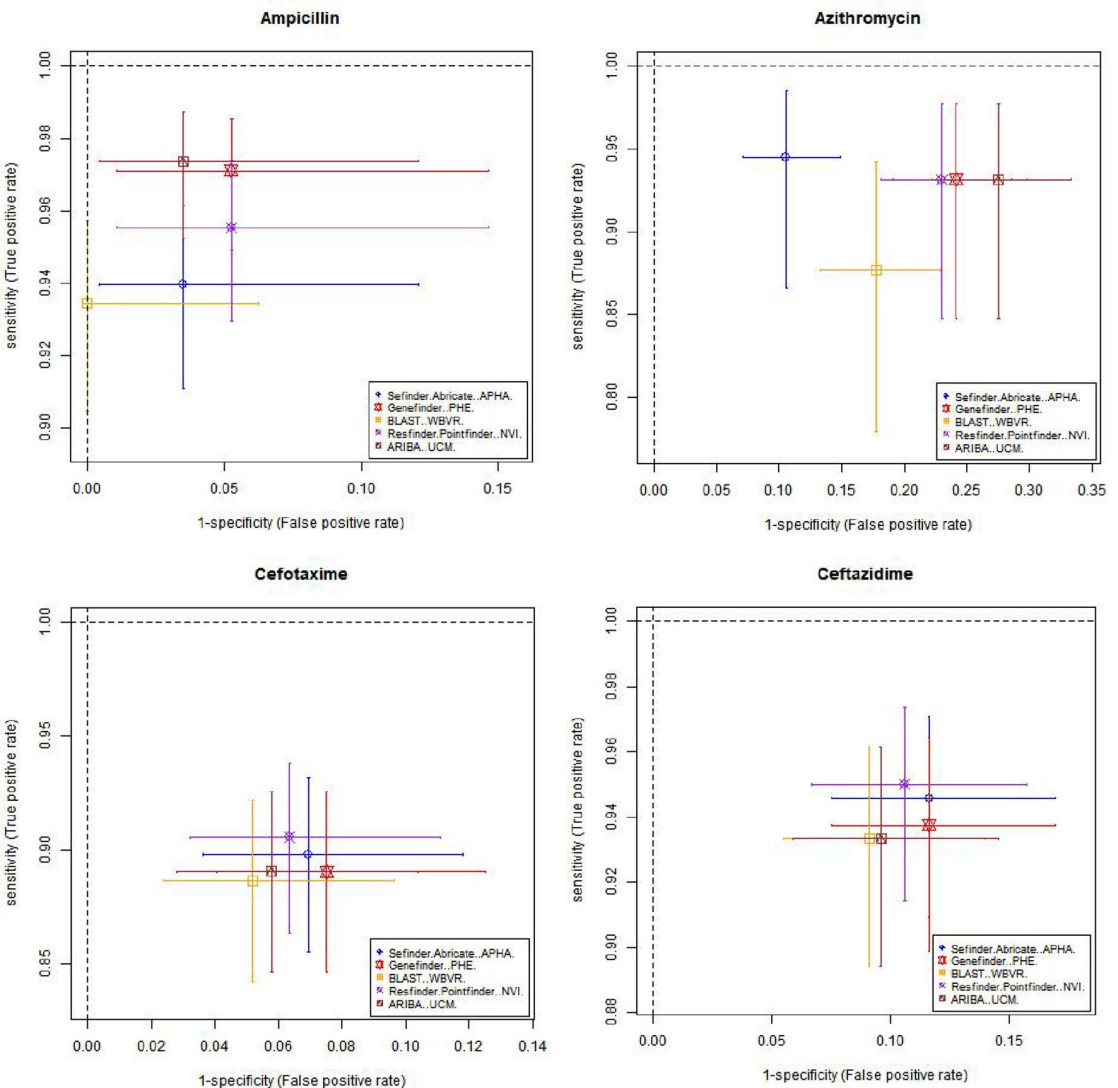

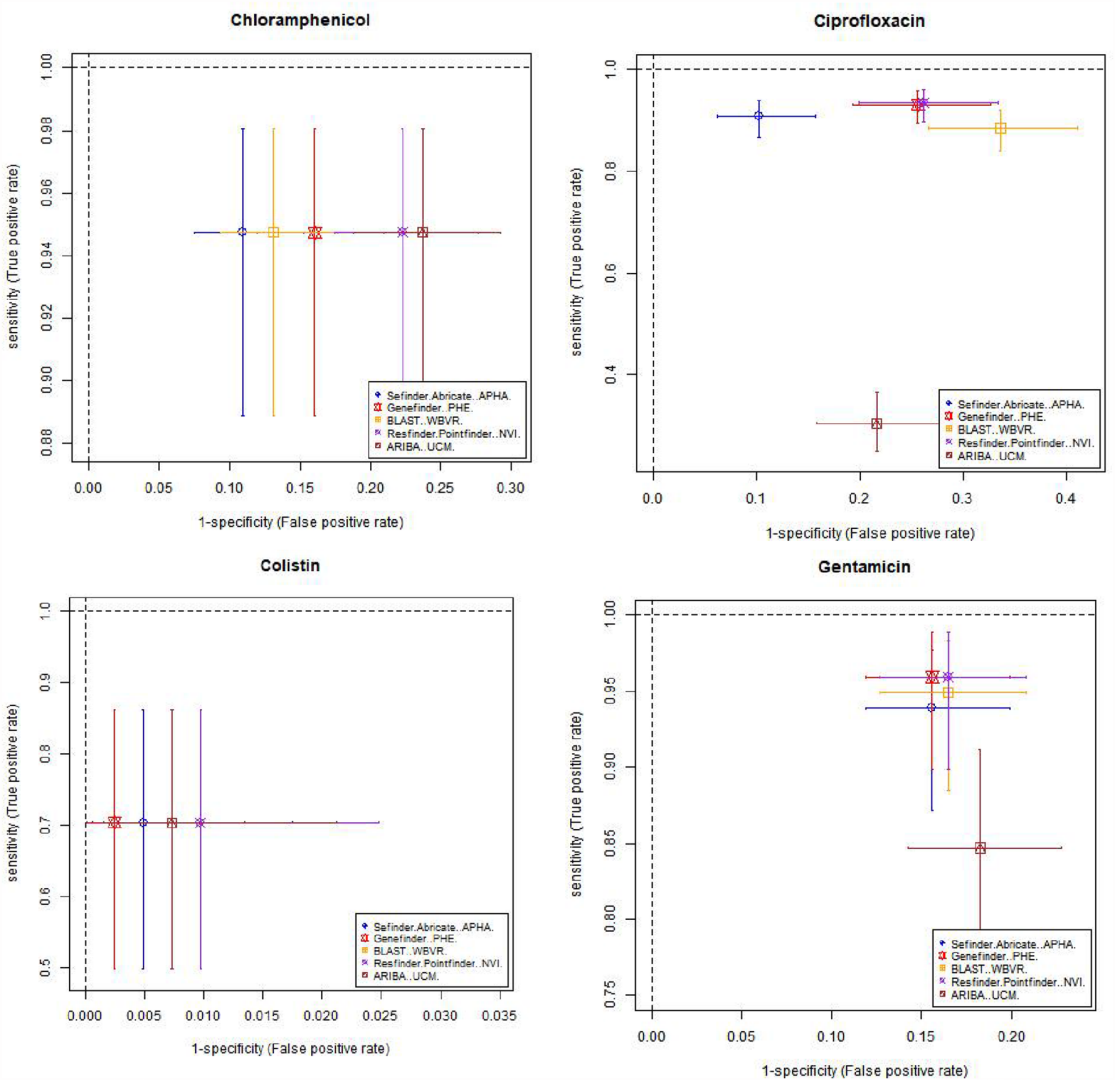

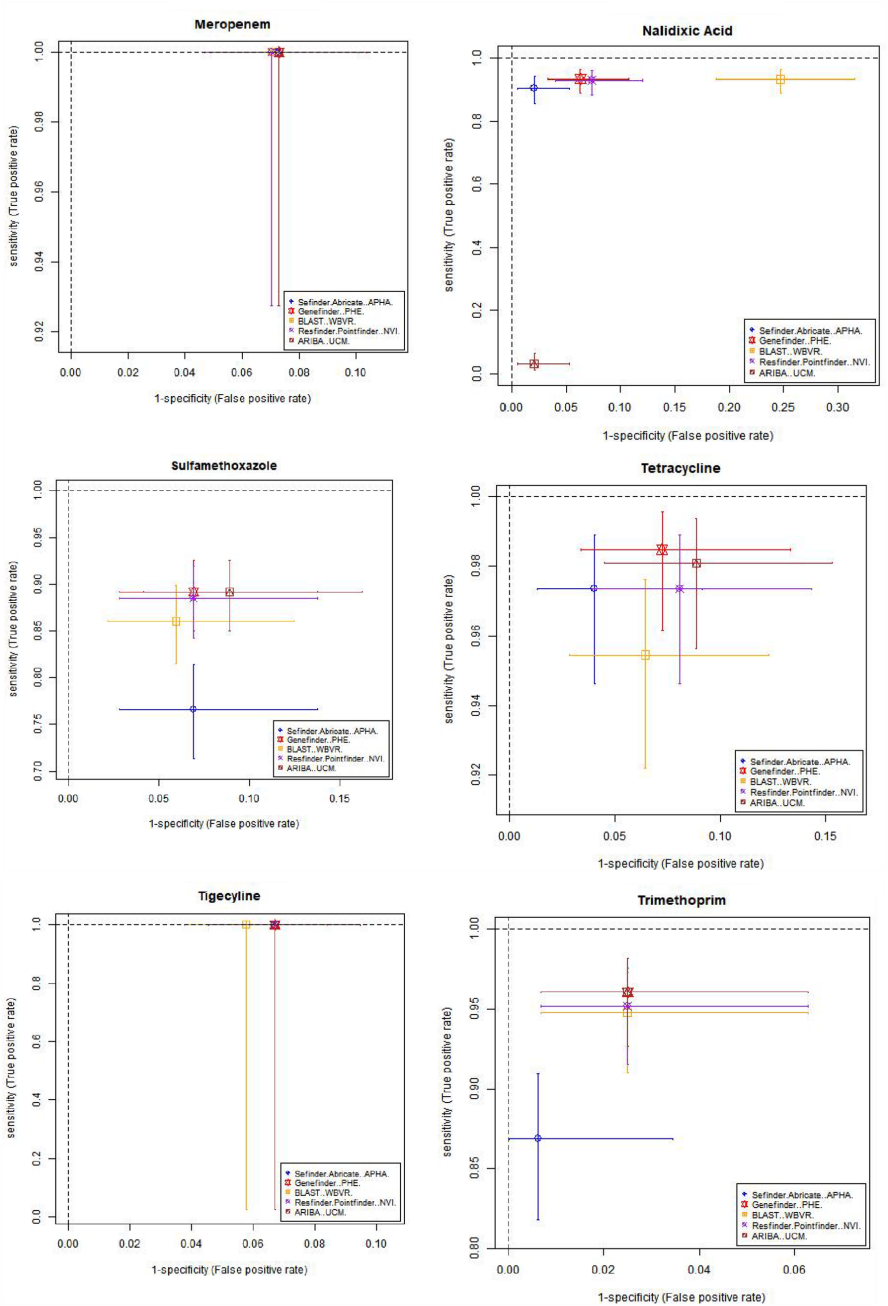
Graphical representation of the sensitivity and specificity values (and 95% confidence intervals) for each antimicrobial detected by the 5 pipelines on the receiver operating characteristic (ROC) coordinate system. As there is one isolate with resistance to tigecycline, its sensitivity value was equal to 1 for all the pipelines.

Table 3 and Fig. 1 shows estimates of sensitivity and specificity for each antimicrobial compared to the phenotypic data per isolate for each pipeline, which was calculated based on the phenotype and AMR gene output from each pipeline (Supplementary Table S3). The cells in Table 3 have been coloured depending on the different levels of performance by each pipeline. This should be interpreted with care as there is significant overlap between the confidence intervals from different pipelines, as demonstrated by Fig. 1, with plots showing the sensitivity, specificity and the 95% confidence intervals for different antimicrobials on the receiver operating characteristic (ROC) coordinate system. All pipelines had an overall sensitivity value between 0.9 and 0.95 when comparing the genotype with phenotype for each antimicrobial class (Table 3A) except for ARIBA, due to this pipeline in the default setting only reporting the presence or absence of acquired resistance genes and not including resistance associated with chromosomal point mutations that reduces susceptibility to antimicrobials such as fluroquinolones^25^.The average specificity value, when comparing the genotype with phenotype for each antimicrobial class for all pipelines, (Table 3B) was around 0.89, except for the APHA Seqfinder/ABRicate pipeline that showed a slightly higher value at 0.93 due to the combination of a discovery stage (APHA SeqFinder) and a validation stage (ABRicate). From the sensitivity and specificity estimates, all the pipelines, in general terms, showed similar levels of performance.

**Table 3:**
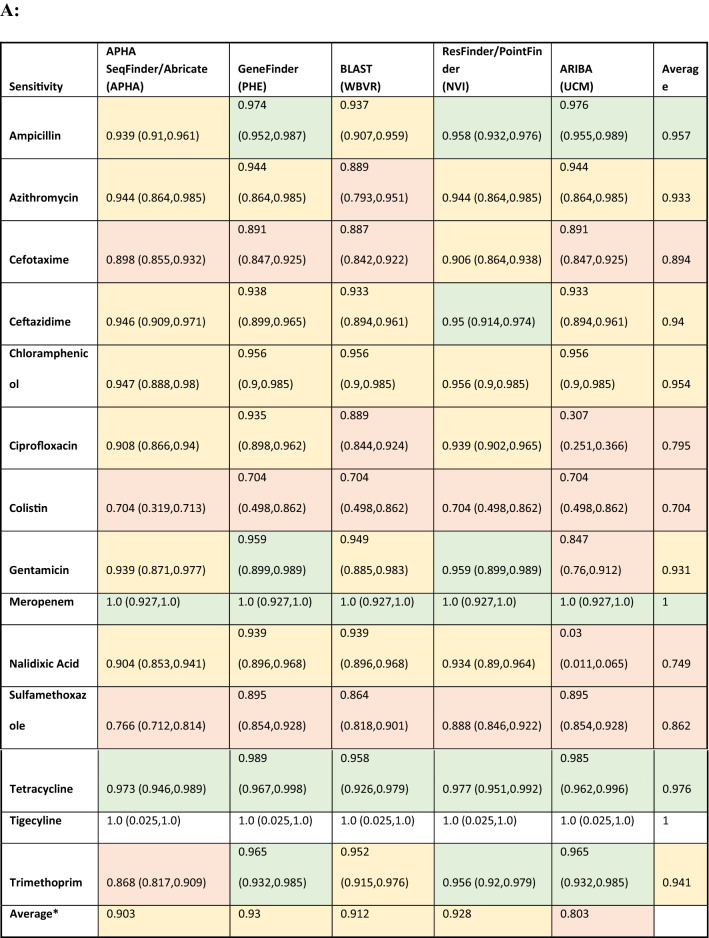

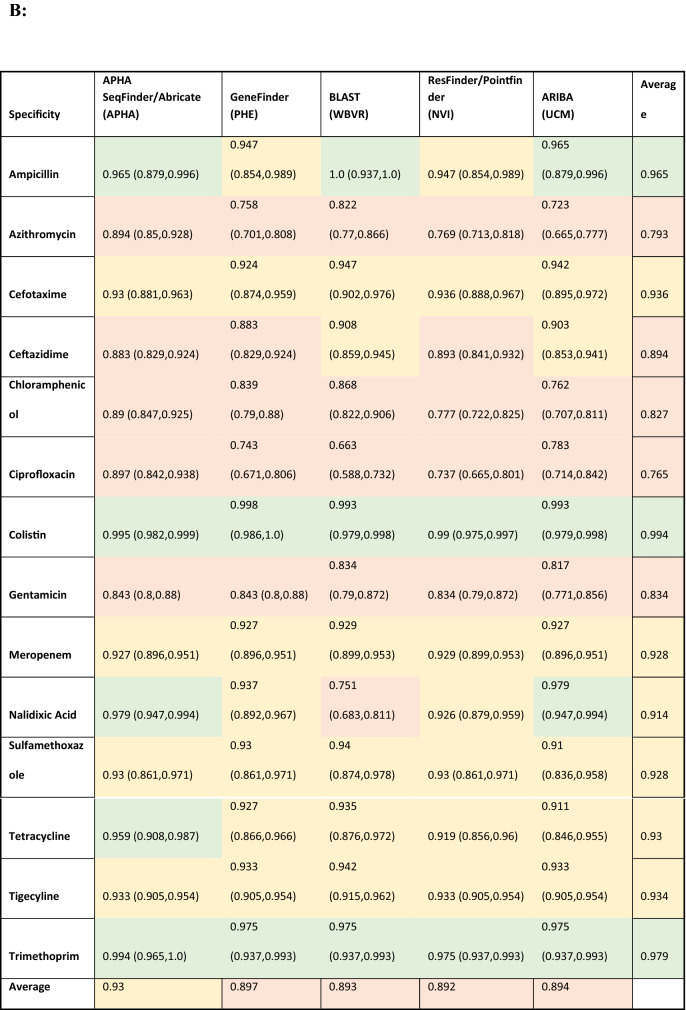
Estimated sensitivity and specificity for each pipeline for each antimicrobial.

The sensitivity (A), specificity (B) values and their 95% confidence intervals (values between brackets) for each pipeline and each antibiotic, with the overall average per pipeline also provided. Cells with values greater than or equal to 0.95 have a green background, between 0.90 and 0.49 orange have background, and less than 0.9 have a red background. The name of the institute that ran the pipeline is given within brackets. *The average values did not include sensitivity for tigecycline.

Resistance to several antimicrobials were easier to detect, such as to ampicillin and tetracycline, with average sensitivity/specificity values equal to 0.95/0.96 and 0.97/0.93, respectively (Table 3 and Fig. 1). Colistin resistance was the most difficult to detect with average sensitivity/specificity values of 0.70/0.99 (Table 3 and Fig. 1). This difficulty can be attributed to two possible factors: the genotype associated to the colistin AMR phenotype might not be fully represented in the database, i.e., there may be an unknown gene or chromosomal mutation that has not yet been associated to this phenotype; and/or the thresholds used in the pipelines for AMR gene detection might be suboptimal for this antimicrobial. Commonly used thresholds for AMR detection in some pipelines include the percentage of an AMR gene present within the isolate and the percentage of similarity between the reference AMR gene and the test isolate. By decreasing or increasing the thresholds, it will be possible to trade off the sensitivity and specificity values, and hence to adjust the detection capabilities. Relaxing the pipeline thresholds will increase the sensitivity and decrease the false negative rate (type II error) causing a decrease of the specificity and the consequent increase of the false positive rate (type I error). Thus, being able to input the pipeline thresholds may be an important feature to adjust the balance between sensitivity and specificity values. Further, this adjustment should be individually defined for each of the antimicrobial classes represented in the database, since a set of thresholds that may be optimal for the detection of one antimicrobial resistance might produce poor results for another. The GeneFinder pipeline was the only pipeline in this study that allowed the user to set individual similarity threshold for each database entry.

Optimum threshold values might also depend on the specific purpose for detecting resistance. In some cases, a very high sensitivity may be preferred as a trade-off to lowering the specificity. For example, more relaxed thresholds for colistin, which belongs to the HP-CIA list^32^, may be used to minimise the occurrence of undetected resistance.

### Interpretation of pipeline results

While performance of the pipeline is the major factor when deciding which one is most suitable to user needs, the ease of interpreting the results files is also an important point to consider. To this end, a questionnaire was completed by the person from each institute responsible for extracting the pipeline output information for their corresponding isolates. The questionnaire contained seven subjective questions to measure the degree of user friendliness related to the interpretation of the pipeline output, and the score for each question and pipeline, given in response by the operator in each institute, are provided in Supplementary Table S4. The average and standard deviation values for responses from all nine institutes, as well as those from the six institutes with no link to any of the software are shown in Table 4. There were no major differences found between the pipelines, with the APHA SeqFinder/ABRicate having the highest average mark and ARIBA the lowest. The preferred pipelines (as per question 7) was GeneFinder by a small margin over APHA SeqFinder/ABRicate when all responses were considered, although the differential was greater when response from only the six “independent” institutes were considered, with ResFinder performing equally well in the latter group with APHA SeqFinder. Therefore, differences in levels of user-friendliness i.e. the ease of finding and linking geno- and pheno-types to understand results, or availability of QC metrics, should also be considered as part of any pipeline harmonisation process, as it may influence who and how often tools are used.

**Table 4:** Questionnaire, average scores and standard deviation (between brackets) for responses from the 9 collaborators for each of the pipelines evaluated (top line), and responses from the six institutes independent from any software used in this study (bottom line).

(Range 1 to 5. 1 being the lowest score and 5 the highest score)	APHA SeqFinder_Abricate	Genefinder	BLAST	ResFinder/PointFinder	ARIBA
1. First impressions when you open the results table	4.22 (0.63)	4.0 (1.25)	4.22 (0.92)	3.33 (0.94)	2.11 (0.87)
	4.33 (0.81)	4.17 (1.33)	3.83 (0.98)	3.33 (1.21)	2 (0.63)
2. How easy was it to find your results in the output file?	4.44 (0.5)	4.44 (0.83)	4.33 (0.82)	4.0 (0.94)	2.56 (0.83)
	4.5 (0.55)	4.5	4.0	3.83	2.5
3. How easy did you find it to link the gene/mutation to your phenotype?	4.06 (0.6)	3.89 (0.87)	4.0 (1.25)	3.78 (0.79)	2.78 (1.31)
	3.92 (0.66)	3.83 (0.98)	3.67 (1.51)	3.83 (0.98)	2.5 (1.64)
4. How easily can an individual non-related to the subject understand the outputs?	3.44 (0.83)	3.0 (0.94)	3.67 (1.05)	3.33 (0.82)	1.78 (0.63)
	3.67 (0.82)	3.17 (1.17)	3.33 (1.21)	3.5 (0.84)	1.83 (0.75)
5. Availability of QC metrics such as mean coverage, etc.	4.78 (0.63)	4.22 (1.23)	1.22 (0.63)	3.11 (1.1)	4.0 (1.25)
	4.67 (0.82)	4.33 (1.21)	1.0 (0)	3 (1.41)	3.83 (1.47)
6. Time used to extract the information	4.11 (0.87)	4.22 (1.03)	3.78 (1.03)	3.44 (1.07)	2.44 (1.17)
	4.17 (0.98)	4.5 (0.84)	3.33 (1.03)	3.33 (1.21)	2.33 (1.51)
7. What is your preferred pipeline?	3.78 (0.63)	4.11 (1.2)	3.44 (0.96)	3.67 (1.05)	2.33 (0.94)
	3.83 (0.75)	4.5 (0.55)	3.17 (0.98)	3.83 (1.17)	2.33 (0.82)
Average score	4.12	3.98	3.52	3.52	2.57
	4.15	4.14	3.19	3.52	2.48

Scores range from 1, being the lowest (worst) score to 5 being the highest (most positive) score.

## Discussion

Standardisation of any methodology is essential to enable comparison, as well as reproducibility across different sectors and countries, but can often be undervalued or overlooked. Monitoring systems that are harmonised already exists in areas such as AMR, which has been invaluable to determine AMR trends overtime across Europe although it only uses phenotypic testing results^33^. The wealth of data WGS provides and the increased cost effectiveness of NGS technology has presented genomic epidemiology as a feasible alternative. However, the availability and continual development of new bioinformatics tools has resulted in a call for harmonisation of in-silico genomic methods to track AMR globally^17^. Recommendations made in a workshop to implement WGS for surveillance recognised the challenges facing its implementation, including some of the bioinformatics processes^20^, which was the focus of this study. Although some of the participating institutes for this study extensively use their preferred in-silico methodologies for AMR detection, in addition to classical wet-lab techniques, the global harmonisation of phenotype-genotype AMR susceptibilities is still in its early stages, due to a lack of pre-set standards. Here we compared several AMR bioinformatics pipelines using the same isolate data set, with default (generalised) pipeline settings and under the same interpretation conditions. Using these conditions, we concluded that no pipeline clearly stands out from the rest, in terms of performance and ease of output interpretation although some user preferences were noted from our questionnaire. Further, we observed that the performance of the pipelines depended in some instances on the antimicrobial for which the resistance determinant was being detected and therefore the ability to set individual thresholds for each database entry is an important feature but not widely available.

We believe that the results of our study can be applied to inform future initiatives for harmonisation of results from WGS pipelines, whether for AMR or any other area of diagnostics and surveillance. Just as multi-locus sequence typing of *E. coli* using underlying genetics^7^ is increasingly being used in place of serotyping^34^ to identify pathogens due to its ability to provide more detailed/accurate subtyping of populations, we believe a harmonised WGS method will do the same for bacterial characterisation, including for AMR. Therefore, our recommendations for harmonisation are as follows. Firstly, it may not be relevant which pipeline is used as long as it verifies a certain level of performance that can be agreed by the relevant scientific experts, depending on the application and the establishment of common inclusion/exclusion criteria of targeted matches. We propose that a control set of isolates are used to test and evaluate any pipeline with an appropriate representative sample and pre-set validation thresholds. For example, the collection of isolates used in this study may be appropriate for testing in-silico AMR pipelines, although any well validated set may be included, provided there is a balanced ratio between resistance and susceptibility of isolates to antimicrobials included in the test panel, e.g., to the EFSA panel of antimicrobials. However, the isolate panel will need to be regularly updated to incorporate isolates with new/novel AMR genes and the pipelines re-evaluated. However, for AMR pipelines, different bacterial species such as MRSA or *Brachyspira*, which may have different AMR mechanisms, a control set of isolates representative of AMR in those species will need to be included. The pipeline database will require to be updated to include species specific AMR genes/mutations and thresholds for these resistance determinants evaluated. For ease of evaluation and interpretation of pipeline results we recommend different species be tested separately using the same principles as performed in this study.

Also, a certain level of pre-agreed performance in terms of minimum sensitivity and specificity thresholds, when comparisons are made between phenotypes and genotypes, should be used as a validation test for any AMR detection software. From the results of this study a sensitivity and specificity value of ~ 0.9, would be reasonable to use, although, for detecting resistance to HP-CIAs e.g. colistin or carbapenem, a more relaxed threshold may be used to maximise resistance detection, including of new gene variants. Secondly, we recommend unifying the databases used by different pipeline software for positive identification; unless genes present in databases, including their nomenclature, are harmonised, there will be differences in the output even from the same isolate test set. In the AMR context, AMR gene identifiers or sequences, including any chromosomal point mutations leading to reduced susceptibility, and the translation rules from genotype to phenotype, should be consistent and transparent. This will also help the naïve user in interpretation of genotypic data, in addition to promoting harmonisation. And thirdly, to allow greater access and usability of this technology for routine surveillance, the final output information should be standardised into user-friendly documents. This will enable individuals with minimal background in genetics to benefit from these softwares.

As there are countless bioinformatics tools available, and many of them pursue similar aims but use different approaches with numerous fine tune adjustments, continual comparison of their performance is a difficult task. Our recommendation to achieve harmonisation does not require focusing on the best performing software, but on setting a common evaluation process based on universal minimal performance thresholds e.g. sensitivity and specificity measures applied to a representative testing sample set. In other words, we have made recommendations which will help towards creation of an appropriate structure for global standardisation of the bioinformatics component to enable genomic surveillance and diagnostics to become routine and standardised worldwide.

## Methods

### The isolates

A total of 436 *E. coli* isolates were provided by nine European institutes: the French Agency for Food, Environmental and Occupational Health and Safety, Lyon France (49 isolates), the Universidad Complutense de Madrid, Spain (50 isolates), the Institute Pasteur, France (50 isolates), the German Federal Institute for Risk Assessment, Germany (50 isolates), the Norwegian Veterinary Institute, Norway (50 isolates), the Wageningen Bioveterinary Research, The Netherlands (50 isolates), the University of Surrey, United Kingdom (50 isolates), the Animal and Plant Health Agency, United Kingdom (37 isolates) and Public Health England, United Kingdom (50 isolates).

The raw WGS reads of isolates, generated from Illumina sequencing described elsewhere^16^, are available in the NCBI nucleotide archive under project number PRJNA805266.

### The antimicrobials

The sensitivity of isolates to the 14 antimicrobials used for AMR monitoring by the European Food and Safety Authority, was assessed using a standard MIC protocol^35^. The antimicrobials were: Ampicillin, Azithromycin, Cefotaxime, Ceftazidime, Chloramphenicol, Ciprofloxacin, Colistin, Gentamicin, Meropenem, Nalidixic Acid, Sulfamethoxazole, Tetracycline, Tigecycline and Trimethoprim. The susceptibility of wild type *E. coli* to the panel were categorised as sensitive (S) or resistant ( R) by: Sensitive, when the isolate was inhibited at an antimicrobial concentration equal or lower than the established ECOFF value for the MIC, as described by the European Committee on Antimicrobial Susceptibility Testing (EUCAST)^36^ ; and Resistant, when the isolate was not inhibited at a specific antimicrobial concentration higher than the established ECOFF values^36^. The full S and R profiles for each isolate to the panel of antimicrobials, interpreted using ECOFFs, are provided in Table S1 and the total values in Table 2.

For some institutions MIC values were not available for part or all isolates for an antimicrobial, this has been marked with an asterisk in Table 2. In most cases these were for human samples for antimicrobials which are not routinely screened by PHE (e.g. azithromycin, chloramphenicol, sulfamethoxazole, tetracycline and trimethoprim).

### Detection software

Description of the five AMR detection software used in this study are provided below:

**GeneFinder**. Public Health England (PHE), UK^28^. **URL**: https://github.com/phe-bioinformatics/gene_finder. **Version**: 2.7. **Operator**: PHE. **Language**: python 2.7.5. **Input format**: FASTQ. **Algorithm**: mapping (bowtie 2.1.0). **Reference database**: provides three in house references sets in FASTA format for *E. coli, Salmonella* and *Campylobacter*. Users can incorporate their own reference set. **Reference database used in this study**: in house (based on institute knowledge, ResFinder database (updated 10.02.2020) and CARD (The Comprehensive Antibiotic Resistance Database, https://card.mcmaster.ca). The database is provided with the tool. **Detection**: presence or absence of sequences and mutations. It also reports insertions, deletions, mixed positions and large indels. Possibility to set the similarity thresholds (between sample DNA and a reference DNA) individually for each gene. **Quality metrics**: coverage, similarity, depth and coverage distribution.

**APHA SeqFinder/ABRicate**. Animal and Plant Health Agency (APHA), UK^14^. **URL**: https://github.com/APHA-AMR-VIR/APHASeqFinder**Version**: 3.0. **Operator**: APHA. **Language**: python 3. **Input format**: FASTQ. **Algorithm**: mapping (smalt 0.7.6). **APHA SeqFinder Reference database**: provides three in house reference sets in FASTA format for AMR genes, mutations, plasmids, virulence factors and heavy metal resistances. **APHA SeqFinder Reference database used in this study**: in house (based on institute knowledge, ResFinder database [updated 10.02.2020] and CARD). **Detection**: presence or absence of sequences and mutations. **Quality metrics**: coverage, similarity, depth and normalised depth by MLST genes. ABRicate^19^ is used in conjunction with SeqFinder as an additional filter. **URL**: https://github.com/tseemann/abricateVersion: 0.7. **Language**: perl. **Input format**: FASTA assembled contigs. (SPAdes 3.13.1). **Algorithm**: BLAST 2.7.0 or higher. **ABRicate Reference database**: same reference database as used for APHA SeqFinder (see above); it also provides additional databases which were not used in this study. **Detection**: presence or absence of genes. **Quality metrics**: coverage and similarity.

**BLAST**, Wageningen Bioveterinary Research (WBVR), The Netherlands. Pipeline not published at the time of this study. **Operator**: WBVR. **Input format**: FASTA assembled contigs. **Algorithm**: raw reads are error corrected with Tadpole from the BBduk suite v38.71. Quality trimming to Q20 with BBduk. Genomes are assembled using SPAdes 3.13.1. Assemblies are compared to the reference database using BLAST version 2.9.0. (with filters: 98% sequence identity and 97% gene coverage). **Reference database**: ResFinder database (updated 10.02.2020). **Reference database used in this study**: ResFinder database (updated 10.02.2020). **Detection**: presence or absence of sequences and mutations. **Quality metrics**: sequence identity and gene coverage provided by BLAST.

**ResFinder v.3.2 + PointFinder v.3.1.0**^29,30^ Technical University of Denmark.

**URL**: https://bitbucket.org/genomicepidemiology/resfinder/src/master/. **Operator**: The Norwegian Veterinary Institute (NVI). **Language**: python 3. **Input format**: FASTQ or FASTA assembled contigs. **Algorithm**: BLAST is used to analyse assemblies (FASTA files). Mapper KMA is used to analyse read data (FASTQ files). **Reference database**: ResFinder database (updated 10.02.2020) and PointFinder_database. **Reference database used in this study**: ResFinder database (updated 10.02.2020) . **Detection**: presence or absence of sequences and mutations. **Quality metrics**:

**ARIBA v2.12**^31^, Sanger Institute, UK. **URL**: https: //github.com/sanger-pathogens/ariba. **Operator**: Universidad Complutense de Madrid (UCM). **Language**: python 3. Input format: FASTQ. Algorithm: mapping (Bowtie 2.1.0). **Reference database**: does not provide its own reference database but has an integrated method to download and standardise one from different sources such as CARD, ResFinder, ARG-ANNOT, MEGARes, NCBI, PlasmidFinder, VFDB, SRST2 and VirulenceFinder. Users can incorporate their own reference set. **Reference database used in this study**: ResFinder database (updated 10.02.2020)). **Detection**: presence or absence of AMR sequences only (This is the default setting and was used in this study. However it is possible to incorporate an external reference database for detecting mutations, but currently there is not an integrated and standardised database for mutations conferring AMR).It also reports genetic fragmentations, interruptions, and duplications. **Quality metrics**: gene coverage, sequence identity.

### Data analysis

All 436 isolates were run through each pipeline by five independent operators (one per pipeline). Result tables from the five pipeline runs were sent to each of the nine institutes who extracted the results corresponding to their isolates. For the following pipelines the antimicrobial class associated with each resistance gene was provided in the output to enable matching with the phenotype by operators: ResFinder; APHA SeqFinder; GeneFinder; WBVR Blast; for ARIBA prior knowledge from operators was required. The AMR genotype information (genes or chromosomal mutations) for each isolate was collated with the gold standard phenotype on a standardised form for each antimicrobial (Supplementary Table S3).

A bespoke R script was used to calculate the sensitivity and specificity and their 95% confidence intervals for each pipeline, for each antimicrobial, by using the information provided in Supplementary Table S3. For a specific pipeline-antimicrobial-isolate combination, if an AMR element was detected, the isolate was considered resistant to that antimicrobial from that pipeline (test positive). If no AMR element was detected the isolate was considered sensitive (test negative). Test results were then compared to the gold standard resistant/sensitive phenotypic profiles.

### Pipelines output evaluation questionnaire

A questionnaire to evaluate user friendliness and quality control metrics of the pipelines output documents was sent to the 9 people, one at each institute, that were responsible for extracting the information for each of the 5 pipelines for their corresponding isolates. Three of the responses were from APHA, PHE and WBVR, who were also running their own pipelines, APHA Seqfinder, GeneFinder and BLAST; but both tasks were not carried out by the same person. The other six responses were from institutes with no link to any of the software used in the study, and an additional evaluation was performed on this subset.

## Supplementary Information

Supplementary Information 1.

Supplementary Information 2.

Supplementary Information 3.

Supplementary Information 4.

## Data Availability

All WGS data is available through NCBI BioProject ID: PRJNA805266.
