# Supplementary material for: Harmonisation of in-silico next-generation sequencing based methods for diagnostics and surveillance
Source: Sci Rep. 2022 Aug 23;12:14372. doi: 10.1038/s41598-022-16760-9 (PMC9396611; doi:10.1038/s41598-022-16760-9)
Supplement: Supplementary file 2 — Supplementary Information 2. [file 41598_2022_16760_MOESM2_ESM.docx]

| **Software** | **GeneFinder** | **APHA SeqFinder/ABRicate** | **BLAST** | **ResFinder/PointFinder** | **ARIBA** |
| --- | --- | --- | --- | --- | --- |
| **Origin** | Public Health England | Animal and Plant Health Agency /  T. Seemann (University of Melbourne) | Wageningen  Bioveterinary Research | Technical University of Denmark | Sanger Institute |
| **Version** | 2.7 | APHA SeqFinder 3.0  ABRicate 0.7 | 2.9.0 | ResFinder 3.2  PointFinder 3.1.0 | 2.12 |
| **Data Input format** | WGS FASTQ | WGS FASTQ/Assembly FASTA | Assembly FASTA | WGS FASTQ or Assembly FASTA | FASTQ |
| **Algorithm for gene detection** | Mapping against reference database | Mapping against reference database /  BLAST against reference database | BLAST against reference database | Mapping against reference database /  BLAST against reference database | Mapping against reference database |
| **Reference database options** | In house or external. | In house or external | External | In house | External |
| **Reference** database **used** | In house. Associated to GeneFinder 2.7 (based on user knowledge/ResFinder database updated 10.02.2020/ CARD*) | In house. Associated to APHA SeqFinder 3.0.0 (based on user knowledge /ResFinder database updated 10.02.2020& CARD*) | Associated to ResFinder database updated 10.02.2020 | Associated to ResFinder database updated 10.02.2020 | Associated to ResFinder database updated 10.02.2020 |
| **Detection** | Genes and point mutations | APHA SeqFincer: Genes and point mutations. ABRicate: only genes. | Genes and point mutations | Genes and point mutations | Genes under default settings as was used in this study (but it is possible to include an external reference database for detecting point mutations) |

**Supplementary Table S2**. Description of the different AMR software used.

* The Comprehensive Antibiotic Resistance Database (https://card.mcmaster.ca/).
